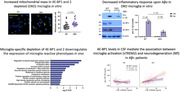# The mTOR‐4E‐BP1 axis controls microglia inflammatory and neurodegenerative responses

**DOI:** 10.1002/alz.087688

**Published:** 2025-01-03

**Authors:** Sara Bermudez, Junghyun Choi, Sunghoon Kim, Jacob W. Vogel, Niaz Mahmood, Vivian Yuchan Zhu, Moein L Yaqubi, Jo Anne Stratton, Oskar Hansson, Argel Aguilar Valles, Luke M Healy, Nahum Sonenberg

**Affiliations:** ^1^ McGill University, Montreal, QC Canada; ^2^ Stony Brook University, Stony Brook, NY USA; ^3^ Department of Clinical Sciences Malmö, SciLifeLab, Lund University, Lund Sweden; ^4^ University of British Columbia, Vancouver, BC Canada; ^5^ Memory Clinic, Skåne University Hospital, Malmö Sweden; ^6^ Clinical Memory Research Unit, Department of Clinical Sciences, Lund University, Lund Sweden; ^7^ Carleton University, Ottowa, ON Canada

## Abstract

**Background:**

Activation of the mTOR pathway is pivotal for microglia to induce and sustain neuroprotective functions (Ulland et al., 2017; Wang et al., 2022). mTOR complex 1 (mTORC1) inhibits the translation repressors, eukaryotic translation Initiation Factor 4E (eIF4E)‐Binding Proteins (4E‐BPs), via phosphorylation, which causes their release from eIF4E to promote mRNA translation (Hay and Sonenberg, 2004). mTORC1 promotes mitochondrial biogenesis via inhibition of 4E‐BPs, by preferentially stimulating the translation of mitochondria‐related mRNAs (Gandin et al., 2016; Morita et al., 2013). We investigated the mechanisms at the intersection of 4E‐BP‐dependent translational regulation and metabolism in microglial response to soluble Aβ.

**Method:**

We carried out immunoblot analysis to investigate the phosphorylation status of 4E‐BP1, the isoform most abundant in microglia, following exposure to Ab. We manipulated the mTOR pathway by knocking out the downstream effectors, 4E‐BPs, to alleviate translation suppression in microglia *in vitro* and *in vivo*. We crossed the microglia‐specific 4E‐BPs knockout mouse with a RiboTag mouse to pull‐down ribosome‐bound mRNAs, providing a genome‐wide pool of actively translating mRNAs in the absence or presence of 4E‐BPs. Finally, we examined the relationship between 4E‐BP1 levels and neuroinflammation markers in cerebrospinal fluid (CSF) of AD patients.

**Result:**

We showed that 4E‐BP1 is inhibited acutely upon exposure to soluble Ab, which is dependent on Spleen Tyrosine Kinase (SYK) activation upstream of mTORC1, but is reduced upon chronic exposure. Furthermore, 4E‐BP1 expression is induced during prolonged exposure to Ab. The deletion of 4E‐BPs in microglia *in vitro* leads to an increase in mitochondrial mass and reliance on oxidative phosphorylation while decreasing expression of pro‐inflammatory mediators and cell death upon exposure to Ab. We observed that increased levels of 4E‐BP1 in the CSF of patients with Aβ pathology are associated with higher neurodegeneration (Nfl) in the presence of microglial activation.

**Conclusion:**

We demonstrate that mTORC1 signaling critically impacts microglia physiology and promotes neuroprotective functions via 4E‐BP1 inhibition. 4E‐BP1 activity in microglia engenders a dysfunctional or detrimental state that may lead to increased neurodegeneration. Therefore, 4E‐BP1 is an attractive target for microglia modulation in AD.